# How Staying Negative Is Good for the (Adult) Brain: Maintaining Chloride Homeostasis and the GABA-Shift in Neurological Disorders

**DOI:** 10.3389/fnmol.2022.893111

**Published:** 2022-07-08

**Authors:** Kelvin K. Hui, Thomas E. Chater, Yukiko Goda, Motomasa Tanaka

**Affiliations:** ^1^Department of Developmental and Molecular Biology, Albert Einstein College of Medicine, Bronx, NY, United States; ^2^Institute for Aging Research, Albert Einstein College of Medicine, Bronx, NY, United States; ^3^Laboratory for Synaptic Plasticity and Connectivity, RIKEN Center for Brain Science, Wako, Japan; ^4^Synapse Biology Unit, Okinawa Institute for Science and Technology Graduate University, Onna, Japan; ^5^Laboratory for Protein Conformation Diseases, RIKEN Center for Brain Science, Wako, Japan

**Keywords:** potassium chloride cotransporter-2 (KCC2), Na^+^-K^+^-2Cl^–^ cotransporter-1 (NKCC1), neuropsychiatric disorders (NPD), neurodevelopmental disorders (NDD), chloride homeostasis, GABA-shift

## Abstract

Excitatory-inhibitory (E-I) imbalance has been shown to contribute to the pathogenesis of a wide range of neurodevelopmental disorders including autism spectrum disorders, epilepsy, and schizophrenia. GABA neurotransmission, the principal inhibitory signal in the mature brain, is critically coupled to proper regulation of chloride homeostasis. During brain maturation, changes in the transport of chloride ions across neuronal cell membranes act to gradually change the majority of GABA signaling from excitatory to inhibitory for neuronal activation, and dysregulation of this GABA-shift likely contributes to multiple neurodevelopmental abnormalities that are associated with circuit dysfunction. Whilst traditionally viewed as a phenomenon which occurs during brain development, recent evidence suggests that this GABA-shift may also be involved in neuropsychiatric disorders due to the “dematuration” of affected neurons. In this review, we will discuss the cell signaling and regulatory mechanisms underlying the GABA-shift phenomenon in the context of the latest findings in the field, in particular the role of chloride cotransporters NKCC1 and KCC2, and furthermore how these regulatory processes are altered in neurodevelopmental and neuropsychiatric disorders. We will also explore the interactions between GABAergic interneurons and other cell types in the developing brain that may influence the GABA-shift. Finally, with a greater understanding of how the GABA-shift is altered in pathological conditions, we will briefly outline recent progress on targeting NKCC1 and KCC2 as a therapeutic strategy against neurodevelopmental and neuropsychiatric disorders associated with improper chloride homeostasis and GABA-shift abnormalities.

## Introduction

The predominant form of neurotransmission in the brain occurs at chemical synapses where presynaptic neurons release neurotransmitters that bind to receptors on postsynaptic neurons. A major type of postsynaptic receptors are ion channels which allow the selective influx or efflux of monovalent (Na^+^, K^+^, and Cl^–^) or divalent (Ca^2+^) ions into and out of postsynaptic neurons. For any particular ion, the direction of flow is dictated by the electrochemical gradient, and the difference in concentrations of ions across the membrane results in a membrane potential. In the adult brain, the opening of ion channels permeable to cations such as Na^+^ and Ca^2+^ drives the membrane potential toward the threshold potential (depolarization) through their entry into the cell. Increased permeability for Cl^–^ anions and their cell entry, in contrast, hyperpolarizes the cell to drive the membrane potential further away from the threshold potential, and reduces the chance of the cell firing an action potential.

The primary inhibitory neurotransmitter in the central nervous system (CNS), γ-aminobutyric acid (GABA), activates ionotropic GABA_A_ receptors that are permeable to chloride, and the maintenance of intracellular and extracellular chloride concentrations ([Cl^–^]_i_ and [Cl^–^]_e_, respectively) is crucial for effective GABAergic neurotransmission. The intracellular chloride concentration ([Cl^–^]_i_) relative to its extracellular concentration is generally lower in the mature brain than in the immature brain, and thus the opening of GABA_A_ receptors results in the influx of chloride ions into the postsynaptic neuron and hyperpolarizes it (Fukuda et al., 1998; Kuner and Augustine, 2000). Since disruptions in the excitation-inhibition (E-I) balance has been strongly linked to a number of neurological disorders such as epilepsy and autism spectrum disorder (ASD) (Fritschy, 2008; Nelson and Valakh, 2015), and inhibition critically depends on chloride levels, there has been much research interest in recent years to determine whether and how impairments in chloride homeostasis contribute to these pathologies.

**FIGURE 1:**
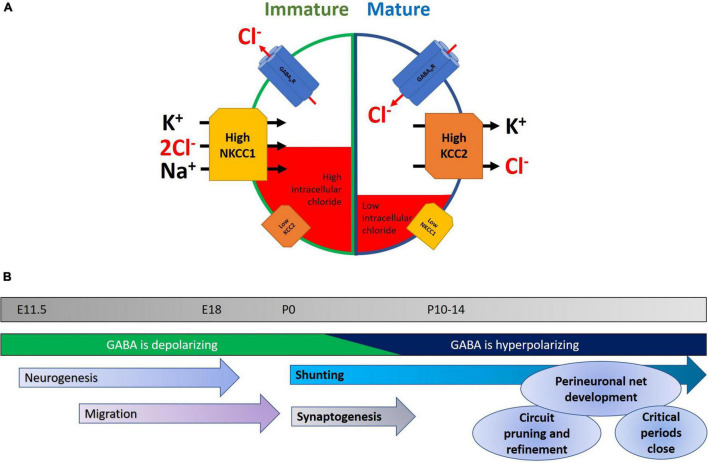
**(A)** Developmental changes in NKCC1 and KCC2 within neurons. In immature neurons, NKCC1 is expressed at high levels and is stabilized at neuronal plasma membranes, where it transports chloride into the cell, along with potassium and sodium. A concomitant low level of KCC2 expression keeps intracellular chloride levels high. The net result of this is that opening of GABA_A_ receptors results in an efflux of chloride, leading to neuronal depolarization and a higher probability of neuronal activity. Later in development, the GABA-shift in neurons is primarily characterized by an upregulation of KCC2, potentially accompanied by a downregulation of NKCC1. This mature state is characterized by lower levels of intracellular chloride, and consequently chloride enters the cell upon GABAergic activity, and hyperpolarizes the neuron, reducing the likelihood of action potential generation. **(B)** Timeline highlighting some key events during early development. Normal development of the nervous system is characterized by a series of stages in which neurons are born, migrate to their final location, and then begin to make synapses. Early in the program, depolarizing GABA helps in aspects of neurogenesis, migration, and elevating activity in the nascent network. As the GABA-shift switches GABA to being hyperpolarizing, this coincides with the onset of an increase in glutamatergic synaptic drive. Later in development, interneuronal activity and structure continue to be refined with the formation of perineuronal nets and the closure of critical periods.

Neuronal chloride homeostasis is maintained primarily by K^+^-Cl^–^ cotransporter-2 (KCC2) (Jarolimek et al., 1999; Kakazu et al., 1999; Rivera et al., 1999; Hübner et al., 2001b) and Na^+^-K^+^-2Cl^–^ cotransporter-1 (NKCC1) (Kakazu et al., 1999; Lu et al., 1999), which act to export and import Cl^–^ out of and into the cell, respectively. Because cells normally have a high [K^+^]_i_, KCC2 is able to transport the chloride ion (along with K^+^) against its concentration gradient in order to maintain a low [Cl^–^]_i_ (Payne et al., 1996). Similarly, NKCC1 uses the electrochemical gradient of Na^+^ to move K^+^ and Cl^–^ into the cell (Gamba et al., 1994; Figure 1A).

During the development and maturation of the nervous system, intracellular chloride levels in neurons change dramatically in concentration, from 37 mM at E18, to 12 mM at P16, however it should be noted that such measurements are not trivial (Owens et al., 1996; Arosio and Ratto, 2014). This initial higher [Cl^–^]_i_ in immature neurons causes a chloride efflux when GABA_A_ receptors are activated and ultimately results in membrane depolarization, thus effectively making GABA signaling excitatory in nature in the immature nervous system. While depolarizing responses are typically excitatory, there are counter-examples of GABA acting to depolarize cells leading to inhibition of network activity (for example see Kirmse et al., 2015). During early postnatal neurodevelopment, changes in expression of KCC2 and NKCC1 are believed to reduce the intracellular chloride concentration, thereby causing GABAergic neurotransmission to become inhibitory (Kakazu et al., 1999; Rivera et al., 1999; Yamada et al., 2004). In human neocortices, their expressions reach adult levels between postconceptional week (PCW) 40–50 (but see Sedmak et al., 2015 for an example of continuing postnatal increases), while in rodent hippocampus and cortex these changes primarily occur during the second week following birth (Rivera et al., 1999; Dzhala et al., 2005). The drop of [Cl^–^]_i_ was largely attributed by earlier studies to the downregulation of NKCC1 in neurons as they mature (Plotkin et al., 1997; Hübner et al., 2001a; Yamada et al., 2004), although the exact expression pattern of NKCC1 still remains somewhat controversial (Virtanen et al., 2020). Subsequent studies have revealed that KCC2 upregulation, in particular the KCC2b isoform, likely plays a prominent role for the observed GABA-shift in the cortex during brain maturation (Rivera et al., 1999; Dzhala et al., 2005; Zhu et al., 2005; Uvarov et al., 2007). This is complicated, however, by observations of functionally inactive KCC2 in immature neuronal populations related to subcellular localization, oligomerization, and phosphorylation status (Balakrishnan et al., 2003; Khirug et al., 2005; Blaesse et al., 2006), thus suggesting that expression alone may not necessarily imply transporter function. Despite some controversies surrounding their specific contributions to the GABA-shift during brain development, KCC2 and NKCC1 both act to maintain a low intracellular chloride level in mature neurons, and thus ensure inward Cl^–^ flow upon GABA_A_ receptor activation.

## The Effects of GABA During Development

### Diverse Roles of GABA Prior to the GABA-Shift

Before discussing the GABA-shift in details, let us first describe the diverse roles of GABAergic signaling during normal neurodevelopment. The transition of highly plastic young brain into the relatively stable adult state involves multiple sets of overlapping processes (Figure 1B). These include the laying down of perineuronal nets, the buildup of extracellular matrix (Hensch, 2005), the refinement of excitatory and inhibitory neuronal networks, and the topic of this review: the change of GABA acting as a depolarizing, excitatory neurotransmitter to a hyperpolarizing inhibitory one. It is worth paying attention to the different roles demanded of the mature and immature nervous system, and how GABA acts on each in turn. In the developing nervous system, depolarizing GABA promotes calcium entry into cells (Leinekugel et al., 1995), which in turn triggers various signaling cascades leading to neurite outgrowth and synapse development (see below). Moreover, excitatory GABAergic synaptic activity combines with glutamatergic inputs to drive waves of activity called giant depolarizing potentials (GDPs) that can then shape synaptic connectivity (Ben-Ari et al., 1989). Notably, deficits in GDPs have been linked to neuropsychiatric disorders (Griguoli and Cherubini, 2017).

Interestingly, the GABA-shift is still retained in brain-derived organoids (Zafeiriou et al., 2020) and appears to be a widespread and robust phenomenon in developing neuronal systems across species. It has not been confirmed that the GABA-shift occurs in humans, however there is immunohistochemical evidence suggesting that human cortical GABAergic development follows a similar course (Pinto et al., 2010).

GABA signaling is active in the developing neuronal network prior to the formation of synapses (Andäng et al., 2008; Wang and Kriegstein, 2008). Following the first wave of synaptogenesis, synaptic GABA responses have been detected before birth in both hippocampus and cortex prior to the formation of excitatory inputs in both rat and mouse tissues (LoTurco et al., 1995; Owens et al., 1996, 1999; Demarque et al., 2002; Gozlan and Ben-Ari, 2003). At the time of birth, GABAergic inputs into CA1 neurons are formed before glutamatergic ones, and appear as the apical dendrite arborizes (Tyzio et al., 1999).

During these early stages, depolarizing GABA signals modulate multiple temporally-overlapping aspects of network development, including the migration of young neurons (Behar et al., 1996, 1998, 2000; Denter et al., 2010; see Barker et al., 1998; Luhmann et al., 2015 for review) to proliferation (both promoting or inhibiting proliferation dependent on the cell type/brain region, see Haydar et al., 2000 for examples of both). Once the components of the network are in place, then depolarizing GABA acts to drive neurite outgrowth, and subsequently promote synapse formation. An early study demonstrated that mouse neuroblastoma cultures treated with GABA had an increase in neurite length and branching (Eins et al., 1983) and this has been repeated across a diverse range of cell types (reviewed in Sernagor et al., 2010). The capacity for GABA to promote neurite development in the cortex (Cancedda et al., 2007) has been shown to depend on chloride homeostasis as inhibiting NKCC1 with the loop diuretic bumetanide reduces dendritic arbor complexity (Wang and Kriegstein, 2011) and furthermore knockdown of NKCC1 slows the development of dendritic arbors (Young et al., 2012). Similarly, GABA has been demonstrated to drive dendritic arborization in the hippocampus (Gascon et al., 2006; Ge et al., 2006; Duveau et al., 2011). Critically, the potentiating effects of GABA on neurite complexity are limited to depolarizing GABA and no longer observed after the GABA-shift (Maric et al., 2001), which suggest that the delayed or extended GABA-shift may result in overgrowth in neuronal arbors and a state of hyperconnectivity.

Earlier work on the depolarizing nature of GABA in young tissue was somewhat controversial (see Holmgren et al., 2010; Zilberter et al., 2010; Mukhtarov et al., 2011; Dzhala et al., 2012; reviewed in Bregestovski and Bernard, 2012; and rebutted in Ben-Ari et al., 2012) due to potential artifacts associated with *in vitro* slice preparations and the difficulty of making *in vivo* measurements of [Cl^–^]_i_. A recent optogenetic approach, where ChR2 was expressed in interneurons (Valeeva et al., 2016), strengthens the case that GABA can be directly depolarizing in the early network. In this study, *in vitro* stimulation of ChR2-expressing interneurons drove a marked increase in EPSC frequency, whereas the same stimulation *in vivo* in anesthetized animals led to a small decrease in EPSC frequency. Nonetheless, even in young neurons, GABA may still act to reduce postsynaptic activity by shunting excitatory currents (Khalilov et al., 1999; Wells et al., 2000), further complicating the picture.

Finally, the network undergoes a large burst in synapse formation during the final stages of depolarizing GABA, with GABA-dependent excitation acting to facilitate the generation of new synapses, both in the hippocampus and cortex (Ben-Ari et al., 1997; Griguoli and Cherubini, 2017), by helping relieve the Mg^2+^ block of NMDARs. Locally applied GABA alone is sufficient to generate new synapses during development (Oh et al., 2016). On the contrary, Salmon et al. (2020) found that blocking GABA_A_ receptors immediately prior to the GABA-shift (in mouse organotypic hippocampal slice cultures) increases the number of glutamatergic synapses formed. However, the authors also assessed the effect of bumetanide inhibition of NKCC1 on excitatory synapse numbers; they found that even under these conditions, GABA inhibition still increased excitatory synaptic density.

Altogether, the downstream effects of GABA on aspects of brain organization and function differ across the GABA-shift. Let us next discuss how the beginning and end of this process is controlled.

### Timing of the GABA-Shift

When exactly does the GABA-shift happen? Interestingly, the exact timing of the switch from depolarizing to hyperpolarizing GABA varies substantially across neuronal type, species, brain region, and even sexes. The shift happens earlier in cortex than in hippocampus (Murata and Colonnese, 2020), earlier and longer-lasting in females than in males (Kyrozis et al., 2006; Nuñez and McCarthy, 2007; Galanopoulou, 2008; but reversed in cerebral cortex, see Roux et al., 2018), and earlier in GABAergic neurons compared to pyramidal cells (although this is also controversial, see Banke and McBain, 2006; Sauer and Bartos, 2010 for opposing examples). Presumably, the regulation of NKCC1 and KCC2 expression pattern that are unique to each experimental context being examined (cell type, brain region, animal sex, and species) contribute to variations in the shift of the actions of GABA.

Throughout the developing nervous system, spontaneous endogenous waves of activity play a profound role in shaping the network connectivity and function. For example, in rodents, eye opening happens over a brief period of one to two days during the second week of life, at a time when the visual network is already functional. Does the depolarizing nature of GABA in the young brain help this process? Interestingly, a recent study (Ge et al., 2021) demonstrated a role for retinal waves in guiding the formation of direction-selective visual network by simulating a naturalistic pattern of optic flow. This occurred at a similar developmental period (P8–11) to the GABA-shift during which rodent retina are either undergoing the GABA-shift (Vu et al., 2000) or have just finished it (Zhang et al., 2005). Previous work in retina has demonstrated also that the switch from depolarizing to hyperpolarizing GABA coincides with the end of the propagating retinal waves, with a role for the regulation of KCC2 (Vu et al., 2000; Sernagor et al., 2003). On the contrary, however, another study has found that intravitreous injection of a GABA_A_ blocker (bicuculine) from birth to P15-18 did not prevent the development of direction-selective circuitry (Sun et al., 2011), raising questions about the necessity of depolarizing GABA for the proper visual network development.

As we have discussed above, given the highly variable timing of the GABA-shift, it is perhaps not surprising that the experimental evidence is mixed as to the identity of the signals that determine the start and end of the GABA-shift. Using rat hippocampal cultures, one study found that blockade of GABA_A_ receptors prevented the increases in KCC2, and thus delayed the GABA-shift (Ganguly et al., 2001); whilst another study, using mouse cultured neurons and organotypic slices, found no contribution of GABA_A_ signaling to the changes in KCC2 (Ludwig et al., 2003). These studies broadly agreed on some points, namely that blocking excitatory neurotransmission (using APV and CNQX) had no effect on the course of the GABA-shift, and that blockade of neuronal spiking with tetrodotoxin (TTX) did not inhibit the GABA-shift. Notably, Khirug et al. (2005) reported that cultured mouse hippocampal neurons showed a step-like change in KCC2 activity after two weeks in culture between DIV 13 and 14, whilst acute slices showed instead a gradual increase from P5 to 14.

In some preparations, neuronal activity does seem to be a critical part of the GABA-shift mechanism. In turtle retina, spontaneous activity begins well before hatching, and the end of spontaneous activity corresponds with KCC2 upregulation (Sernagor et al., 2003). Moreover, dark-rearing turtles delays the GABA-shift, with a correspondingly weaker KCC2 expression. Further work in turtle retina has demonstrated that at least in this model, GABA_A_ activity is required for the switch (Leitch et al., 2005). Similar to the study by Ganguly et al. (2001), the chronic blockade of GABA_A_ signaling in turtle retina prevented the switch. On the contrary, a study using mouse retina found that the GABA-shift normally could be detected between P7-9, which was complete by P9-11, and that mouse retinal explants cultured in blockers of GABA_A/B/C_ signaling still underwent the GABA-shift (Barkis et al., 2010). In fact, the same study subsequently blocked nearly all activity using a combination of drugs including TTX and glutamatergic signaling and still observed the GABA-shift.

What could be the triggers of the GABA-shift? Barkis et al. (2010) found that retinal ganglion cells (RGCs) cultured alone failed to make the switch. Importantly, the impaired switch was rescued by co-culturing with cells from the superior colliculus, suggesting a diffusible factor originating from other cell types as a required component, while the possible contribution of brain-derived neurotrophic factor (BDNF) could be excluded. On the contrary, the BDNF precursor pro-BDNF has been shown to regulate KCC2 levels (Riffault et al., 2016), keeping it low during development. Intriguingly, BDNF itself, either exogenously applied (Ludwig et al., 2011) or using a BDNF-overexpressing mouse model (Aguado et al., 2003), increases KCC2 levels despite KCC2 levels being unaffected in *Bdnf* knockout (KO) mice (Puskarjov et al., 2015) (see below for further discussion on BDNF-dependent signaling that regulates KCC2 and NKCC1). Liu et al. (2006) have identified a role for cholinergic signaling in controlling the GABA switch in chick ciliary ganglion, where spontaneous nicotinic cholinergic activity modulates transporter levels, and thus the balance of Cl^–^ across the membrane. Taken as a whole, the evidence largely points to the regulation of KCC2 as being the primary driver behind the onset of the GABA-shift, but the identities of the upstream signal and the basis for cell-type specificity remain to be fully elucidated.

Another example of a potential molecular player in triggering the GABA-shift is the oxytocin receptor, activation of which promotes cell surface expression of KCC2 and stabilizes it at the cell membrane (Leonzino et al., 2016). Neurons from oxytocin receptor KO mice show a delayed GABA-shift, which nevertheless fully completes a few days later than control animals. Additionally, in these cultures NKCC1 levels were not different in KO cells, again consistently with KCC2 being the key player (Leonzino et al., 2016). It is worth mentioning here that there is another, transient GABA-shift during childbirth, that is also triggered by oxytocin, and acts to protect the fetus against anoxic episodes (Tyzio et al., 2006). Interestingly, the target for this transient GABA-shift has been suggested to be in fact NKCC1 activity and not KCC2, thus demonstrating that under certain conditions NKCC1 can dramatically influence GABA-shift behavior. It is worth mentioning that other candidates have been proposed to explain the suppression of neuronal activity during birth (for example vasopressin, see Spoljaric et al., 2017)].

Finally, another aspect of the GABA-shift concerns the developmental change in the GABA_A_ receptor activity itself. GABA_A_ receptors are large pentameric channels composed of a mixture of α, β, and γ subunits, often accompanied by the accessory δ and ρ subunits (Hevers and Lüddens, 1998). An early study that characterized GABA_A_ receptor subunit mRNA levels in rat brain tissue found a change in the α subunit at around P8, from α2/3 to α1 in several brain regions (Laurie et al., 1992). Similarly, immunohistochemical studies suggest that α1 expression ramps up following birth and is still increasing at around the time of the GABA-shift (Fritschy et al., 1994; Davis et al., 2000) with a concomitant decrease in α2 levels. Importantly, GABA_A_ receptors containing α1 subunits are more sensitive to GABA than those containing α2/3 (Wafford et al., 1993; Ebert et al., 1994; Hevers and Lüddens, 1998) and as such, the switch in the receptor subunit composition may serve to exaggerate the GABA-shift.

## Cell Signaling Pathways and Mechanisms Affecting KCC2 and NKCC1 Expression and Function

### Genetic and Epigenetic Regulation of NKCC1 and KCC2 Expression

**FIGURE 2:**
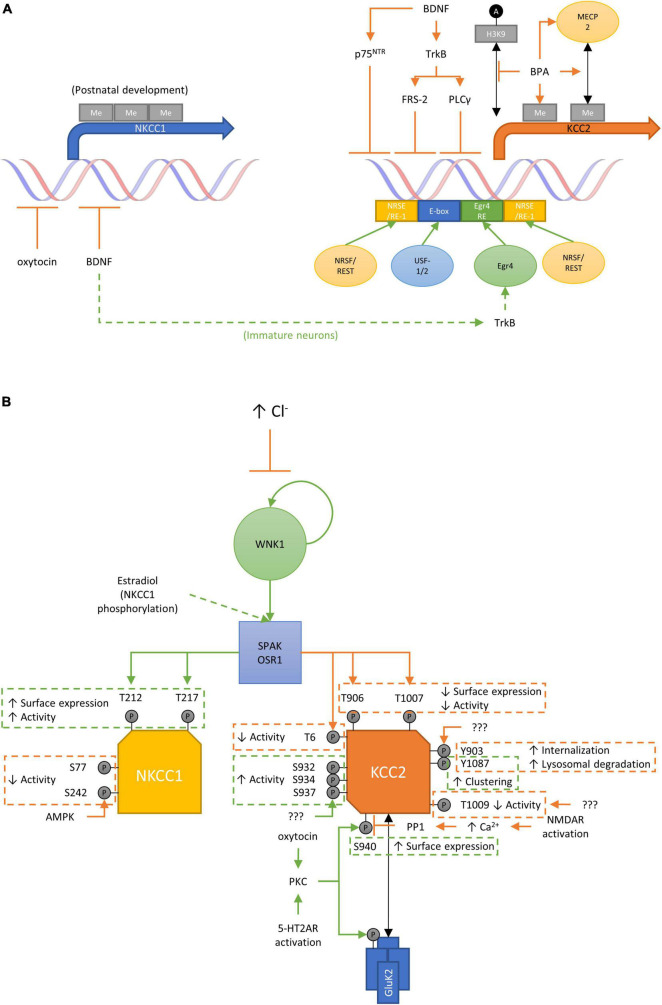
**(A)** Transcriptional and epigenetic mechanisms involved in regulating KCC2 and NKCC1 expression. Transcriptional regulatory elements including NRSE/RE-1, E-box, and Erg4 RE are involved in regulating the expression of KCC2 and NKCC1 by various stimuli such as oxytocin and BDNF-TrkB/p75^NTR^ signaling. Additional regulatory mechanisms including DNA methylation and histone acetylation have also been demonstrated for KCC2 regulation. Given the association of chromatin regulators and other proteins that regulate gene expression with various neurodevelopmental disorders such as ASD, it will be of interest to determine how the expression of KCC2 and NKCC1, and ultimately the GABA-shift, may be affected in those pathologic conditions. A, acetylation; Me, DNA methylation. **(B)** Phospho-regulatory network by WNK/SPAK/OSR1 and other kinases to modulate KCC2 and NKCC1 activity. WNK kinases function both as a sensor and effector of intracellular chloride concentrations as their catalytic activities are influenced by the direct binding of Cl^–^ ions. SPAK/OSR1 are phosphorylated by WNK kinases and in turn can phosphorylate KCC2 and NKCC1 proteins. Numerous phosphorylation sites have been identified on KCC2 and NKCC1, which ultimately modulate their activities directly or by influencing their surface expression or degradation. Other phosphorylation sites have been identified on KCC2 and NKCC1 (discussed in main text) but were found to have no effects on transporter functionality and/or surface expression. P, phosphorylation.

A number of genetic and epigenetic mechanisms regulating KCC2 expression have been identified thus far (Figure 2A). KCC2 is encoded by the *SLC12A5* gene to produce two isoforms (KCC2a and KCC2b), both of which are expressed only in neuronal cells (Payne et al., 1996; Uvarov et al., 2007). This is principally mediated by a 1.4 kb promoter fragment and two RE1-Silencing Transcription factor (REST)/Neuron-Restrictive Silencer Factor (NRSF) repressor elements identified in its 5′ promoter region and the first intron of the KCC2b isoform (Yeo et al., 2009). Notably, the 1.4 kb promoter fragment alone is sufficient to restrict KCC2 expression in neuronal cells (Uvarov et al., 2005), and the fragment contains binding sites for transcription factors such as Early growth response 4 (Egr4) (Uvarov et al., 2006) and Upstream stimulating factors 1 and 2 (USF-1/2) (Markkanen et al., 2008), suggesting these as key factors in controlling neuronal expression.

Given its role in mediating chloride homeostasis and consequently the direction of Cl^–^flow triggered by GABA_A_ receptor signaling, it is not surprising that the regulation of KCC2 expression also depends on the maturation status of the neuron of interest. For instance, as mentioned above, BDNF is known to regulate KCC2 expression. In particular, BDNF enhances KCC2 expression in immature neurons but suppresses it in mature neurons (Rivera et al., 2002, 2004; Aguado et al., 2003; Wake et al., 2007; Shulga et al., 2008; Boulenguez et al., 2010; Ludwig et al., 2011). Such opposing effects appear to be mediated via distinct pathways as BDNF activates expression in immature neurons via the ERK1/2 pathway and Egr4 (Ludwig et al., 2011), whereas its inhibitory effects on KCC2 expression in mature neurons depends on a signaling cascade involving Src homology domain containing transforming protein/FGF receptor substrate 2 (Shc/FRS-2), phospholipase C γ (PLC-γ), and cAMP response element-binding protein CREB (Rivera et al., 2004). The regulation of KCC2 by BDNF is further complicated by the effects of its precursor form (pro-BDNF), which can also suppress KCC2 expression via binding to the p75 neurotrophin receptor (p75^NTR^) (Rivera et al., 2002; Riffault et al., 2016). Thus, the overall effects of BDNF and pro-BDNF on KCC2 expression likely depends on the combinatorial expression patterns of BDNF/pro-BDNF and their respective receptors in the developing brain. Notably, this is further supported by the observations that p75^NTR^ is highly expressed during development (Yang et al., 2014; Menshanov et al., 2015; Winnubst et al., 2015) and that BDNF/pro-BDNF are involved in various activity-dependent changes in neurons (Gubellini et al., 2005; Langlois et al., 2013; Yang et al., 2014).

In contrast, much less is currently known about the regulation of NKCC1 expression (encoded by the *SLC12A2* gene), but both BDNF and oxytocin have been observed to downregulate its expression (Ceanga et al., 2010; Eftekhari et al., 2014; Tyzio et al., 2014). The factors required to maintain high NKCC1 expression in immature neurons however remains to be identified.

In addition, although much less is known about epigenetic regulation of *SLC12A2* and *SLC12A5*, several recent studies have also demonstrated that the expressions of NKCC1 and KCC2 may be modulated via epigenetic mechanisms. Exposure of cultured developing human and rat cortical neurons to Bisphenol A (BPA), a possible chemical toxicant used in the production of a wide range of consumer products linked to increased incidence of neurodevelopmental disorders (Nesan and Kurrasch, 2019), was found to delay the KCC2 expression and increase [Cl^–^]_i_ during neuronal maturation (Yeo et al., 2013). BPA treatment was observed to alter both the methyl-CpG binding protein 2 (MECP2) binding to the KCC2 promoter and its histone H3K9 acetylation. Furthermore, DNA methylation of the KCC2 regulatory region was found to be increased by BPA treatment while the administration of a DNA methyltransferase inhibitor partially rescued the reduction of KCC2 expression caused by BPA. Consistent with other studies using chemical or genetic HDAC inhibition (Yeo et al., 2009; Hou et al., 2018; Ouyang et al., 2019), this study also found *Hdac1/2* knockdown to increase *SLC12A5* mRNA levels and rescue the BPA-induced KCC2 downregulation (Yeo et al., 2013). Conversely, DNA methylation of the NKCC1 promoter region increases significantly during postnatal neurodevelopment and *in vitro* DNA methylation of the NKCC1 promoter region reduces NKCC1 expression (Lee H. A. et al., 2010). Together, these studies point to possible epigenetic mechanisms regulating KCC2 and NKCC1 expression to mediate the GABA-shift during neurodevelopment.

### WNK-SPAK/OSR1 Pathway Regulates KCC2/NKCC1 Functions

Aside from the transcriptional mechanisms described above, KCC2 and NKCC1 are also regulated via post-translational modifications that modulate their surface expression and activity levels. While other kinases have been shown to regulate NKCC1 and KCC2, the with-no-lysine (K) (WNK)-serine/threonine protein kinase 39 (SPAK)/OSR1 oxidative stress-responsive 1 (OSR1) pathway is a principal regulator for both transporters to coordinate their activities based on intracellular chloride concentrations (Alessi et al., 2014; Shekarabi et al., 2017).

Of all four WNK isoforms (WNK1-4) in the human genome, WNK1 is believed to be the principal WNK kinase in the human CNS (Shekarabi et al., 2013). This is supported by the observation that *WNK1* mutations result in an autosomal recessive disease characterized by congenital pain insensitivity (Shekarabi et al., 2008). WNK kinases effectively function as intracellular chloride sensors, through Cl^–^ binding to their catalytic domains in an inhibitory manner that suppress their autophosphorylation and kinase activity (Piala et al., 2014). Active WNK kinases phosphorylate SPAK and OSR1 (Moriguchi et al., 2005), which in turn directly phosphorylate KCC2 and NKCC1 to suppress and enhance their activities, respectively (Pacheco-Alvarez et al., 2006; Vitari et al., 2006; de los Heros et al., 2014; Figure 2B). When [Cl^–^]_i_ is low, chloride-unbound WNK kinases are active and consequently suppress KCC2 (to reduce Cl^–^ export) and increase NKCC1 activation (to enhance Cl^–^ import). In contrast, WNK kinases are inhibited by high [Cl^–^]_i_, thereby reversing these changes to enhance KCC2-mediated Cl^–^ export while suppressing further import by NKCC1.

Specifically, activated SPAK/OSR1 targets threonine residues T906 and T1007 (all residue numbers refer to human sequences) located on the C-terminal intracellular domain of KCC2 (de los Heros et al., 2014), which significantly suppress its Cl^–^ transport activity (Rinehart et al., 2009). Notably, while these sites were partially phosphorylated in neonatal mouse brains, there was no evidence of their phosphorylation from adult brains, thus providing fully active KCC2 to export chloride ions to maintain the inhibitory nature of GABAergic signaling. Moreover, a separate study directly implicated the WNK1-SPAK/OSR1 signaling pathway in modulating KCC2 function and GABA activity by enhancing its phosphorylation at these two sites in immature neurons (Friedel et al., 2015). Specifically, the authors observed that *in utero* knockdown of *Wnk1* resulted in lower [Cl^–^]_i_ and a hyperpolarizing shift for GABA specifically in immature neurons (P3-5), as there were no observable effects on mature neurons (P30). These findings thus support a role for WNK kinases in maintaining the high intracellular chloride concentration necessary for GABA to have depolarizing effects in the immature brain and may potentially be involved in regulating the GABA-shift. As described above, WNK kinases in theory should be inhibited by the high [Cl^–^]_i_ present in immature neurons and in turn reduce KCC2 phosphorylation to enhance Cl^–^ export. However, although *Wnk1* knockdown was found to reduce [Cl^–^]_i_ in immature neurons, Friedel et al. (2015) did observe higher T906 and T1007 phosphorylation on KCC2 in immature neurons compared to their mature counterparts; thus suggesting that either the WNK-SPAK/OSR1 pathway remained sufficiently active despite the high [Cl^–^]_i_ in immature neurons or that additional kinases are at work during this stage. Further studies comparing the WNK-SPAK/OSR1 pathway between immature and mature neurons are necessarily to fully delineate the importance of this pathway to [Cl^–^]_i_ regulation and the GABA-shift during neurodevelopment. More recently, two groups independently produced knock-in mice with phosphomimetic mutations in these two phosphorylation sites (i.e., T906E and T1007E) to evaluate their physiologic significance. The authors observed reduced postnatal survival of homozygous mutants due to respiratory arrest and epileptic seizures (Watanabe et al., 2019), while heterozygotes displayed abnormal ASD-like impairments in ultrasonic vocalization and social behavior (Pisella et al., 2019), thereby demonstrating further the physiological importance of these two phosphorylation sites. Pharmacologically, *N*-ethylmaleimide (NEM) was found to activate KCC2 activity by enhancing the phosphorylation and dephosphorylation of S940 (to be discussed below) and T1007, respectively, to stabilize its surface expression (Conway et al., 2017). It was revealed that NEM mediates its effects on KCC2 by reducing WNK-dependent SPAK phosphorylation (S373) and depends on the dephosphorylation of T1007 on KCC2 as the overexpression of the T1007E phosphomimic mutant abolished the effects of NEM on KCC2 activity. Conversely, experiments using knock-in mice with alanine replacement at these phosphorylation sites demonstrated that they are critical to the timing of the GABA-shift (Moore et al., 2019). Moreover, the S906A/T1007A mutations altered social behaviors and led to slight enhancements in specific cognitive functions (Moore et al., 2019), while also protecting against chemoconvulsant-induced seizures (Moore et al., 2018). In addition, the WNK-SPAK/OSR1 pathway is also known to phosphorylate the N-terminal T6 of KCC2a, which is missing in KCC2b due to alternative splicing of exon-1a (Uvarov et al., 2007; Markkanen et al., 2017). Similar to its C-terminal counterparts (T906 and T1007), phosphorylation of T6 by SPAK was found to reduce its transporter activity (Markkanen et al., 2017).

In contrast, the activity of NKCC1 is enhanced by the phosphorylation of T203, T207, T212, T217, and T230 by the WNK-SPAK/OSR1 pathway (Darman and Forbush, 2002; Flemmer et al., 2002; Kahle et al., 2005; Vitari et al., 2006). Specifically, it was demonstrated that T217 phosphorylation is necessary for NKCC1 activity while the other sites serve modulatory roles (Darman and Forbush, 2002). Thus, through the coordinated phosphorylation of KCC2 and NKCC1, the WNK-SPAK/OSR1 pathway serves to monitor [Cl^–^]_i_ and alter KCC2/NKCC1 activities to maintain an optimal intracellular chloride concentration. This control of KCC2/NKCC1 activity by the WNK-SPAK/OSR1 pathway is primarily achieved by influencing the surface expression of these two cotransporters on the cell membrane (Rinehart et al., 2009).

In addition to modulation by the WNK-SPAK/OSR1 pathway, the activities of KCC2 and NKCC1 are also regulated by other phosphorylation events. The phosphorylation of KCC2 at serine residue S940 is mediated by protein kinase C (PKC) and enhances its surface expression to increase overall transporter activity. The physiological importance of this phosphorylation site was revealed as knock-in mice incapable of S940 phosphorylation (i.e., S940A mutants) exhibit ASD-like deficits in social behaviors and cognitive functions (Moore et al., 2019), while also showing accelerated onset of status epilepticus and lethality from chemical-induced seizure activity (Silayeva et al., 2015). As for its regulatory signaling, both oxytocin and 5-hydroxytryptamine type 2A receptor activation are known to increase KCC2 activity by promoting S940 phosphorylation via PKC-dependent mechanisms (Lee et al., 2007; Bos et al., 2013; Leonzino et al., 2016). Notably, PKC can also indirectly influence KCC2 surface expression by enhancing complex formation between KCC2 and kainate receptors via the phosphorylation of subunit GluK2 (Mahadevan et al., 2014; Pressey et al., 2017; Garand et al., 2019). Conversely, S940 dephosphorylation can be triggered by calcium influx via NMDA receptors through the actions of protein phosphatase 1 (PP1) (Lee et al., 2011).

Aside from these serine and threonine residues, KCC2 tyrosine phosphorylation at Y903 and Y1087 have also been observed by as yet unidentified tyrosine kinases. These C-terminal phosphorylation sites are believed to enhance KCC2 internalization and lysosomal degradation (Lee H. H. C. et al., 2010), as the phosphorylation-incompetent Y903F/Y1087F mutant showed increases in both total and surface KCC2 expression (Lee H. H. C. et al., 2010). Notably, Strange et al. (2000) had originally drew similar conclusions about Y1087 phosphorylation as they observed the phosphomimic Y1087D mutation to completely abolish KCC2 activity in *Xenopus* oocytes without any changes in surface expression of the mutants. It remains unclear, however, how phosphorylated Y1087 could directly impact KCC2 activity. In addition, other groups have also found the Y1087D variant to disrupt KCC2 activity in subsequent studies (Akerman and Cline, 2006; Pellegrino et al., 2011). By contrast, an independent study using the same aspartate substitution at Y1087 but considered it as a non-phosphorylatable mutant concluded that Y1087 phosphorylation activates KCC2 activity by influencing its distribution on the cell membrane and regulating its clustering (Watanabe et al., 2009). Notably, the authors also used genistein or lavendustin A, and sodium vanadate as tyrosine kinase inhibitor and tyrosine phosphatase inhibitor, respectively, to manipulate Y1087 phosphorylation status to examine its effects; it must be taken into consideration that these compounds are non-specific and therefore KCC2 Y1087 phosphorylation-independent effects cannot be dismissed. Given this, and that aspartate (as well as glutamate) substitutions are typically considered as phosphomimics rather than a non-phosphorylatable state, the findings by Watanabe et al. (2009) may in fact be in agreement with the previous studies about the effects of Y1087 phosphorylation even though the precise molecular mechanism underlying its influence on KCC2 function remains largely unknown. Apart from Y903 and Y1087, additional phosphorylation sites at both the N- (S25, S26, S31, and T34) and C-termini (S932, S934, S937, T999, T1009, S1022, S1025, and S1026) have been identified to date (Weber et al., 2014; Cordshagen et al., 2018). Using alanine and aspartate mutants, the authors demonstrated S932D, S934D, S937D, and T1009A substitutions to enhance KCC2 functions, whereas mutations at the other sites had no effects. Importantly, the S932D, S934D, S937D, and T1009A mutations did not influence surface expression of KCC2 and thus suggested that the effects were directly on its transport activity.

As for NKCC1, AMPK has been shown to phosphorylate it at S77 and S242, which in turn suppresses its activity (Sid et al., 2010; Fraser et al., 2014). In both studies, AMPK was shown by both groups to directly phosphorylate N-terminal fragments of NKCC1 *in vitro* using recombinant proteins. While phosphorylation of NKCC1 at these sites appear to be AMPK-dependent, additional studies are necessary to determine whether they occur and are mediated directly by AMPK *in vivo*, especially in the context of neural cells, given the contrasting evidence from these and other groups (Sid et al., 2010; Miraucourt et al., 2016; King et al., 2019). Altogether, the above studies reveal the dynamic regulation of KCC2 and NKCC1 activity at multiple levels by phosphorylation.

KCC2 glycosylation has also been observed on at least six residues (N283, N291, N310, N328, N338, and N339) and are believed to regulate its membrane targeting (Agez et al., 2017). Notably, three KCC2 mutations associated with severe early-onset epileptic encephalopathy (L311H, L426P, and G551D) have been shown to reduce glycosylation and surface expression (Stödberg et al., 2015).

It is clear that the correct expression, surface targeting, and phosphorylation status of NKCC1 and KCC2 are critically important for healthy circuit development and function, but how does this impact the myriad of different cell types in the brain? We will discuss this topic next.

## Interactions Between Different Cell Types During the GABA-Shift

### GABA-Shift and Interneuronal Development

Changes in the balance of chloride across neuronal membranes are not expected to strongly affect postsynaptic neuronal activity unless there is concomitant release of GABA from presynaptic inhibitory neurons. The development of the inhibitory network has been widely studied across brain regions (review by Kubota et al., 2016; Lim et al., 2018; Ferrer and García, 2022), and mistimed development or inappropriate levels of inhibitory neuronal activity has been linked to multiple pathological states, with epilepsy being the primary example, but also schizophrenia and autism (reviewed by Marín, 2012; Chu and Anderson, 2015). The mammalian cortex contains a wide range of inhibitory neurons (Petilla Interneuron Nomenclature Group et al., 2008), and they change in number throughout development and past the end of the GABA-shift (for more details on interneuronal development we refer the reader to Gonchar et al., 2008; Bartolini et al., 2013; Ouellet and de Villers-Sidani, 2014). The activity and development of the interneuronal network is implicated in various aspects of circuit maturation, including ending critical periods (Hensch, 2005) and dynamically modulating axonal pruning and dendritic spine formation (Anderson et al., 1995).

Interneuronal development proceeds in stages, beginning at neurogenesis, followed by migration, and finally embedding into and modulating the circuit (Lim et al., 2018). This process is still ongoing throughout the period of the GABA shift, with some subpopulations of interneurons becoming mature earlier than others. Interestingly, the developmental period of the inhibitory neuronal network is temporally extended in larger animals such as humans, and this may contribute to the high levels of cognitive flexibility exhibited by these creatures, whilst also leaving them susceptible to many of the disease states that arise from imprecise GABAergic activity (Kim and Paredes, 2021).

### Glial Contributions to Chloride Regulation

Currently, one critical missing piece of the puzzle is the contribution of glial cell types to the shift in GABA’s effect. The presence of astrocytes has been shown to increase the speed of the GABA-shift *in vitro* (Li et al., 1998), however it is difficult to repeat this kind of experiment *in vivo*. Expression of NKCC1 and KCC2 can act to regulate neuronal intracellular [Cl^–^] levels, but functions of the network critically depend also on the ionic balance of the extracellular milieu for which astrocytes play a central role, particularly of potassium levels (see Hertz and Chen, 2016; Bellot-Saez et al., 2017 for review). In support of such a role, dysfunction in glial potassium homeostasis has pathological downstream effects on neuronal activity (Robel and Sontheimer, 2016). The KCC2 protein requires a low extracellular potassium concentration to carry chloride out of the cell. There is a substantial body of evidence indicating that astrocytes are largely responsible for the clearance of extracellular K^+^ following bouts of neuronal activity (Hertz, 1965; Ransom et al., 2000; Wang et al., 2012), either by local uptake or redistribution and buffering throughout the gap-junction connected astrocyte network. Astrocytes can also clear GABA from the extracellular milieu through GAT1-3 expression and activity (Boddum et al., 2016), and themselves express GABA receptors (MacVicar et al., 1989; Boisvert et al., 2018). Moreover, there is a wealth of data demonstrating that GABA signals can bidirectionally modulate astrocytic calcium levels (Doengi et al., 2009; Matos et al., 2018; Yu et al., 2018), which in turn controls the release of ATP and other gliotransmitters (Kang et al., 1998; Liu et al., 2004; Serrano et al., 2006). Neuronal activity, extracellular Cl^–^ levels, and astrocytic Cl^–^ have been demonstrated to be dynamically linked, and neuronal activity can trigger the release of Cl^–^ from astrocytes into the extracellular space, where it contributes to subsequent GABAergic activity (Egawa et al., 2013). Astrocytes and GABAergic neurons are known to signal to one another (for examples of interneuron to astrocyte, see Mariotti et al., 2018; Mederos et al., 2021; for astrocyte to interneuron, see Shigetomi et al., 2012; reviewed in Mederos and Perea, 2019) in a manner that appears to be specific to particular classes of GABAergic interneurons (Mariotti et al., 2018; Matos et al., 2018), adding an extra layer of complexity to the system.

NKCC1 is expressed in murine astrocytes (Su et al., 2002) and astrocytic internal Cl^–^ levels remains high throughout development, at around 30–50 mM in cultures (Bekar and Walz, 2002) and brain slices (Untiet et al., 2017). *In vivo* measurements have proven tricky, however (see Arosio and Ratto, 2014 for review). Although NKCC1 levels are largely stable in astrocytes, under various pathological conditions NKCC1 expression can be upregulated, undergoes phosphorylation, and is stabilized at the astrocytic plasma membrane (Jayakumar and Norenberg, 2010). Astrocytes from *Slc12a2* KO mice are unable to regulate their volume in response to a hyperkalemic (75 mM K^+^) challenge (Su et al., 2002), and NKCC1 has been implicated in astrocytic swelling in pathological conditions such as ischemia and brain edema caused by liver dysfunction (Yan et al., 2001; Jayakumar et al., 2008; reviewed in Jayakumar and Norenberg, 2010). Astrocytic NKCC1 is upregulated in response to trauma (Jayakumar et al., 2011) and both inhibiting NKCC1 activity pharmacologically with bumetanide or genetically with an anti-NKCC1 siRNA significantly reduces trauma-induced increase in astrocytic volume. It is worth mentioning that the *Slc12a2* KO mouse is viable (Delpire et al., 1999; Dixon et al., 1999; Flagella et al., 1999); however, the loss of the NKCC1 protein causes deficits in neuronal proliferation (Magalhães and Rivera, 2016) and exacerbates the severity of a mouse model of epilepsy (Hampel et al., 2021), amongst other issues. Whereas neuronal loss of NKCC1 appears to be compensated for at the network level (Sipila et al., 2009), the consequences on glia are much less clear. In adult tissue, NKCC1 has been implicated in astrocytic responses to neuronal potentiation (Henneberger et al., 2020), helping coordinate the withdrawal of fine astrocytic processes from synapses. All of the above indicates that deficits in NKCC1 astrocytic expression, function, or surface trafficking may all have pathological consequences. Nevertheless, experimental evidence using astrocytic-specific knockdown or knockout of NKCC1 are currently lacking. As discussed below, NKCC1 has joined the long-list of proteins whose mutation or absence in humans is linked to diseases (reviewed in Koumangoye et al., 2020), although it remains unclear how the loss of NKCC1 function in the affected individuals may have influenced the GABA-shift during their development. In fact, very little is known about the consequences of disrupted astrocytic NKCC1 expression or function on the GABA-shift, and this is a ripe field for study.

Conversely, how about the role of glia in the case of KCC2? Multiple studies have demonstrated a link between chronic inflammatory pain and KCC2 downregulation in the spinal cord (Coull et al., 2003, 2005; Okada-Ogawa et al., 2015; Tsuruga et al., 2016). Interestingly, the trigger for changes in neuronal KCC2 levels seems to be BDNF that is potentially released from nearby glia. Originally, microglia were thought to be the culprits; however, recent evidence suggests astrocytic signaling to be important for regulating both KCC2 and NKCC1. There is further evidence for a more direct role of astrocytes in modulating KCC2 levels. IL-6 released from astrocytes can elevate BDNF expression, and this in turn triggers downregulation of KCC2 in a model of neuropathic pain (Kitayama et al., 2016). It remains unclear if the source of this BDNF is neuronal or astrocytic, or potentially both.

## GABA-Shift Defects in Neurodevelopmental and Neuropsychiatric Disorders

### Effects of *SLC12A2* and *SLC12A5* Mutations and Chromatin Dysregulation

Due to their critical role in maintaining chloride homeostasis and consequently the GABA-shift during brain development, it is not surprising that a number of studies have revealed changes in KCC2 and NKCC1 in patients with various neurodevelopmental and neuropsychiatric disorders.

Several recent studies have identified mutations in *SLC12A5* from children affected by epilepsy in infancy with migrating focal seizures (EIMFS) (Table 1; Stödberg et al., 2015; Saitsu et al., 2016; Saito et al., 2017). The loss-of-function mutations (L311H, L426P, and G551D as mentioned above) identified by Stödberg et al. (2015), reduce KCC2 surface expression in neurons accompanied by depolarized chloride equilibrium potential (E_Cl_), thereby potentially reducing GABA_A_R-mediated hyperpolarization compared to neurons carrying wild-type KCC2 protein. In a separate study (Saitsu et al., 2016), two of the mutations identified from EIMFS patients (E50_Q93del and M415V) significantly elevated E_Cl_ even though no changes in KCC2 cell surface expression were observed. Electrophysiological recordings demonstrated that the presence of these mutations suppressed the ability of the transporter to extrude chloride from the cell’s interior, and overexpression of E50_Q93del and M415V mutants of KCC2 resulted in elevated intracellular chloride concentrations. Furthermore, another study found a distinct mutation (R952H) in KCC2 from patients from an Australian family with febrile seizures that similarly disrupted chloride homeostasis by reducing KCC2 surface expression and function (Puskarjov et al., 2014). Another group also found the same mutation (R952H) along with R1049C to associate with idiopathic generalized epilepsy (IGE) (Kahle et al., 2014). Notably, the authors detected a reduction of S940 phosphorylation in the mutant proteins, previously demonstrated to enhance KCC2 surface expression by reducing its internalization (Lee et al., 2007). More recently, the R952H and R1049C mutations in *SLC12A5* were also found in ASD (Merner et al., 2015). In addition, the same study identified the R1048W mutation in ASD patients, likely to cause similar disruption in KCC2 expression and function as R1049C due to their proximity. Notably, exome sequencing data suggest that ASD patients are more likely than healthy controls to have synonymous mutations predicted to create or disrupt CpG sites, thereby potentially affecting gene expression via alterations in DNA methylation patterns. Aside from these mutations affecting KCC2, recent studies have also identified multiple *SLC12A2* mutations in patients affected by various neurodevelopmental disorders (Table 1; Delpire et al., 2016; Marchese et al., 2016; Anazi et al., 2017; Macnamara et al., 2019; McNeill et al., 2020; Stödberg et al., 2020). The pathogenic effects of these mutations on NKCC1 expression or functions, however, remain largely unknown.

**TABLE 1:** Mutations in *SLC12A5* and *SLC12A2* identified from patients with neurodevelopmental and neuropsychiatric disorders.

Gene	Mutation	Pathology	Reference
*SLC12A5* (KCC2)	R952H	IGE Febril seizures ASD SZ	Kahle et al., 2014 Puskarjov et al., 2014 Merner et al., 2015 Merner et al., 2015
	R1049C	IGE ASD	Kahle et al., 2014 Merner et al., 2015
	R1048W	ASD	Merner et al., 2015
	L331H	EIMFS	Stödberg et al., 2015
	L426P	EIMFS	Stödberg et al., 2015
	G551D	EIMFS	Stödberg et al., 2015
	E50_Q93del (exon 3, 44 aa deletion)	EIMFS	Saitsu et al., 2016
	A191V	EIMFS	Saitsu et al., 2016
	W318S	EIMFS	Saitsu et al., 2016
	S323P	EIMFS	Saitsu et al., 2016
	M415V	EIMFS	Saitsu et al., 2016
	S748del	EIMFS	Saitsu et al., 2016
	S399L	EIMFS	Saito et al., 2017
	R880L	EIMFS	Saito et al., 2017
	V473I	IGE	Till et al., 2019
*SLC12A2* (NKCC1)	V1026Ffs*2	Seizure-like episodes (multisystem dysfunction)	Delpire et al., 2016
	L863F	MAEP	Marchese et al., 2016
	c.2617-2A > G (cDNA)	ID	Anazi et al., 2017
	Exons 2-7 (22kb deletion)	Kilquist syndrome	Macnamara et al., 2019
	H186Afs*17	Severe global developmental delay	McNeill et al., 2020
	V327A	Cortical dysplasia (multiple congenital anomalies)	McNeill et al., 2020
	N376I	Spastic paraparesis and delay of speech and gross motor development	McNeill et al., 2020
	A379L	ASD and ID	McNeill et al., 2020
	R410Q	ASD and mild ID	McNeill et al., 2020
	W892*	Global developmental delay, autism, motor stereotypy and hypotonia	McNeill et al., 2020
	c.2006-1G > A (exon 13 deleted) c.1431delT (exon 8 frameshift)	ID with encephalopathy	Stödberg et al., 2020

ASD, autism spectrum disorder; EIMFS, epilepsy of infancy with migrating focal seizures; ID, intellectual disability; IGE, idiopathic generalized epilepsy; MAEP, macrocephaly autism–epilepsy phenotype; SZ, schizophrenia; *STOP codon.

Recent studies have also observed altered *SLC12A5* and *SLC12A2* expression in patients. Consistent with their opposing roles in maintaining chloride homeostasis and GABA-shift, whereas NKCC1 protein levels were observed to be increased in patients with temporal lobe epilepsy (TLE) (Sen et al., 2007), *SLC12A5* (KCC2) mRNA expression was found to be downregulated (Huberfeld et al., 2007). In addition, patients with tuberous sclerosis or type IIb cortical dysplasia, both of which are common causes of refractory epilepsy, also displayed changes in KCC2 and NKCC1 expression and levels (Talos et al., 2012; Ruffolo et al., 2016).

As described above, previous studies suggest that the expression of KCC2 and NKCC1 can be regulated via epigenetic mechanisms. Notably, a strong link between disrupted chromatin regulation and neurodevelopmental disorders has been implicated in recent years (Hui et al., 2018). Specifically, mutations in a number of chromatin regulators have been associated with neurodevelopmental disorders such as Rett syndrome (*MECP2*), intellectual disability (*CHD1*, *CHD2*, *CHD7*, *CHD8*, *ATRX*, and *KDM5C*), epilepsy (*CHD1*, *CHD2*, *CHD7*, *CHD8*) (Consortium et al., 2013), and ASD (*MECP2*, *CHD1, CHD2*, *CHD3*, *CHD7*, *CHD8*, *MBD1*, *MACROD2*, *H2AFY*, *ARID1B*, *SMARCC1*, *SMARCC2*, and *JMJD1C*) (reviewed in LaSalle, 2013). Two recent studies found *KCC2* RNA expression to be significantly reduced in Rett syndrome patients (Gogliotti et al., 2018; Hinz et al., 2019). At the protein level, KCC2 levels were reduced in cerebrospinal fluid collected from patients with Rett syndrome without a change in NKCC1 levels, thereby disturbing the KCC2/NKCC1 ratio (Duarte et al., 2013). It will be of particular interest to examine how KCC2 and NKCC1 expression are altered in patients carrying mutations in the other chromatin regulators and determine how potential disruptions of the GABA-shift may contribute to those neurodevelopmental disorders.

### GABA-Shift Abnormalities Due to “Dematuration” in Neuropsychiatric Disorders Such as Schizophrenia, Bipolar Disorder, and Major Depressive Disorder

Recent studies have also identified alterations of NKCC1 and/or KCC2 expression in patient-derived brain samples or animal models of neuropsychiatric disorders such as SZ and bipolar disorder (BD). For example, a significant reduction in KCC2 expression and thus an reducing trend for the KCC2/NKCC1 transcript ratio (as NKCC1 mRNA levels were not different between patients and controls) were observed in the hippocampi of SZ patients, suggesting the possibility of an immature GABA-shift (Hyde et al., 2011). Consistent with this, a separate group also observed reduced protein levels of KCC2 in the dorsal lateral prefrontal cortex of SZ patients (Sullivan et al., 2015). A follow-up study of alternative KCC2 transcripts further identified increased expression of transcript EXON6B (with a new exon 6) in SZ patients but the same transcript was reduced in patients with major depressive disorder (MDD), while other alternatively spliced transcripts examined including AK098371 (truncated KCC2 transcript), EXON2B (with a new exon 2), and ΔEXON6 (with exon 6 deletion) were not found to be different (Tao et al., 2012). In contrast, Morita et al. (2014) identified reduced expression of various alternatively spliced transcripts of NKCC1 (NKCC1b and 1-2a) in SZ patients compared to healthy controls. Although it is unclear how these alternative transcripts may affect overall KCC2 and NKCC1 functions, the studies highlight the potential involvement for differentially spliced KCC2 and NKCC1 transcripts in SZ and MDD pathophysiology.

*SLC12A5* and *SLC12A2* mutations have also been identified in SZ patients, including the R952H mutation in *SLC12A5* described above (Merner et al., 2015). The same group later identified a Y199C mutation in the *SLC12A2* gene specifically in SZ patients but not in ASD nor intellectual disability (ID) patients (Merner et al., 2016). Functional analysis of this NKCC1 mutant revealed an increase in its transporter activity, thus potentially altering chloride homeostasis and consequently GABAergic signaling in affected neurons. In addition, increased expression of OSR1 and WNK3 were detected in SZ patients compared to healthy controls (Arion and Lewis, 2010), thus potentially altering KCC2 and NKCC1 phospho-regulation and ultimately disturbing the GABA-shift. Similar to changes in KCC2 and NKCC1 expression discussed above, it remains to be determined how precisely these alterations are involved in pathophysiology, given that neuropsychiatric disorders do not typically manifest until late adolescence or early adulthood even though the mutations have been present during neurodevelopment.

To this end, it is of interest that several studies have recently identified transcriptomic signatures of “immaturity” in the dentate gyri and prefrontal cortices of adult patients with ASD, SZ, or BD (Gandal et al., 2012; Walton et al., 2012; Hagihara et al., 2014; Murano et al., 2019), suggestive of a reversal of the mature state (“dematuration”). Nevertheless, interpretation of the observations in dentate gyri are complicated by the fact that it is a major site of adult neurogenesis and thus the changes may reflect a difference in the process or the state of developmental maturation rather than signifying dematuration *per se*. Furthermore, multiple studies have also observed immature morphological and functional properties in neurons of SZ animal models or mutant animals exhibiting behavioral deficits relevant to neuropsychiatric disorders (Miyakawa et al., 2003; Yamasaki et al., 2008; Ohira et al., 2013; Shin et al., 2013; Takao et al., 2013; Kobayashi et al., 2014; Zhao et al., 2018; Sawada et al., 2020). Given the importance of the GABA-shift from excitatory to inhibitory during brain development and maturation, any disruptions in this process or even its reversal in the form of dematuration of neuronal phenotypes could potentially have profound effects on brain network activity and contribute to neurological disorders. Interestingly, a state of neuronal dematuration can be induced by different types of stimuli such as inflammation, hyperactivity, and drug treatment (Kobayashi et al., 2010, 2012; Takao et al., 2013). In parallel, various studies have shown that KCC2 and NKCC1 expression, phosphorylation status, or function could be affected by various types of stress (Hewitt et al., 2009; Sarkar et al., 2011; Miller and Maguire, 2014; Corradini et al., 2017; Furukawa et al., 2017; Hu et al., 2017) and may coincide with a disruption of neuronal maturation or the induction of a dematuration process. For example, maternal immune activation (MIA) by the injection of double stranded RNA poly(I:C) was found to alter the KCC2/NKCC1 ratio via a proinflammatory cytokine-dependent mechanism, and consequently delay the GABA-shift (Corradini et al., 2017). An independent study found that a similar MIA effectively abolished the GABA-shift and maintained GABA as an excitatory signal (Fernandez et al., 2018). Interestingly, a NKCC1-specific inhibitor was able to restore the hyperpolarizing actions of GABA in hippocampal slices from poly(I:C)-treated animals, thus highlighting the involvement of disrupted KCC2/NKCC1-mediated chloride homeostasis in the neurological deficits caused by MIA. More importantly, such findings indicate that pharmacologic intervention is possible and could have therapeutic benefits for certain patients with neurodevelopmental and neuropsychiatric disorders that have disturbed GABA-shifts.

## Genetic and Pharmacologic Approaches to Modulate KCC2 and NKCC1 Expression to Rectify Neurological Conditions Associated With Hyperexcitability and Excitatory-Inhibitory Imbalance

In addition to the above study that demonstrated the beneficial effects of NKCC1-specific inhibitor bumetanide, commonly used clinically as a diuretic, it has been shown to be a useful treatment in mouse models of SZ (Kim et al., 2021), Down Syndrome (DS) (Deidda et al., 2015), Rett Syndrome (Banerjee et al., 2016), 22q11.2 deletion (DiGeorge) syndrome (Amin et al., 2017), neonatal epilepsy (Dzhala et al., 2005), and ID (Maset et al., 2021). More recent studies have further identified a novel therapeutic molecule known as ARN23746 (Savardi et al., 2020; Borgogno et al., 2021) and a series of KCC2 expression-enhancing compounds (KEECs) to be effective against the core symptoms exhibited by DS and ASD mouse models (Tang et al., 2019). Whereas ARN23746 targets NKCC1 selectively (thus limiting off-target diuretic side effects exhibited by bumetanide), the KEECs act via distinct cell signaling pathways [activation of the sirtuin 1 (SIRT1) or transient receptor potential cation channel subfamily V member 1 (TRPV1) pathways, or inhibition of fms-like tyrosine kinase 3 (FLT3) or glycogen synthase kinase 3β (GSK3β) pathways] to upregulate KCC2 expression at both mRNA and protein levels. In addition, anti-NKCC1 gene therapy or positive modulation of KCC2 overexpression by IGF1 and oxytocin have also been shown to be useful in treating cognitive deficits in a DS (Parrini et al., 2021) and Rett Syndrome mouse models (Tang et al., 2016; Bertoni et al., 2021), respectively.

Thus far, clinical trials have provided mixed results for the use of the NKCC1 blocker bumetanide to help reduce ASD symptoms in patients. While there have been reports hinting at successes in correcting for some symptoms in open-label pilot studies (Lemonnier and Ben-Ari, 2010; Hadjikhani et al., 2018; Fernell et al., 2021) and randomized double-blind clinical trials (NCT01078714 and NCT03156153) (Lemonnier et al., 2012, 2017; Zhang et al., 2020; Dai et al., 2021), other studies (NCT03715153, NCT03715166, and 2014-001560-35) have failed to show significant differences compared to placebo in their primary outcome measures (Sprengers et al., 2020; Crutel et al., 2021; Georgoula et al., 2022). Similarly, there are contrasting findings from small scale studies which attempt to treat patients with other types of neurodevelopmental and neuropsychiatric disorders such as epilepsy, fragile X syndrome, and schizophrenia using bumetanide (Eftekhari et al., 2013; Lemonnier et al., 2016; Rahmanzadeh et al., 2017; Soul et al., 2021), clearly highlighting the need for larger studies to definitively verify its effects. A particular issue with bumetanide potentially hindering its beneficial effects in these trials is its poor penetration into the CNS. Fortunately, the aforementioned ARN23746 appears to have a better *in vivo* pharmacokinetic profile in mice as compared to bumetanide (Savardi et al., 2020), thus offering a glimpse of hope that ARN23746 and its derivatives may have more potent effects in human patients. Furthermore, in dealing with complex disorders such as ASD and schizophrenia in which the precise pathogenic cause is typically unknown for each patient, pharmacologic strategies such as bumetanide may simply not be effective for every patient since it is likely that only a subpopulation are experiencing E-I imbalance due to a dysregulated GABA-shift. As such, brain organoids derived from patient-specific induced pluripotent stem cells (iPSCs) (Zafeiriou et al., 2020) could be used to screen for patients with pathologic alterations in the GABA-shift to determine whether they are likely going to benefit from NKCC1 inhibitors. Finally, another issue with understanding the potentially negative consequences of disrupted NKCC1 function and its contribution to GABA-shift is the widespread expression of the protein (as well as its relatives like NKCC2, for example), and the resulting broad spectrum of problems that may occur throughout the body when they are inhibited. Thus, continued efforts to identify new therapeutic molecules such as ARN23746 and KEECs which do not have peripheral effects like bumetanide may prove to be fruitful.

## Conclusion

This review has attempted to lay out the changes that occur in GABAergic signaling during early development, with a particular focus on the GABA-shift, both in how it proceeds in healthy tissue, and how it is disrupted in various pathological states. Abnormalities in the progression of the GABA-shift have been linked to several neurodevelopmental and neuropsychiatric disorders. Potential therapeutic targets typically focus on a handful of proteins: the transporters that move Cl^–^ across the neuronal membrane, and regulators of those proteins, such as WNK kinases. The balance of Cl^–^ across the membrane is primarily regulated by KCC2 and NKCC1, and as such, they are prime targets for pharmacological modulation. However, the widespread expression of NKCC1 in other tissues of the body complicates the utility of the pharmacological approach for therapy. Furthermore, our understanding of disturbances in the normal GABA-shift is hampered by the high variability: it happens at different times, in different cell types, that may be intermingled within a particular brain region. Moreover, in human cases with a mutated or missing chloride transporter, it is not clear how much of a patient’s symptoms may be attributable to an abnormal GABA-shift, and how much is related to ongoing network dysfunction. Further research is required to understand the pathological consequences of a mis-timed or incomplete GABA-shift, and treatment options, whilst currently available, are limited.

A better grasp of the factors that control the GABA-shift during development may provide a greater understanding of what happens in the process of dematuration within the adult network. What are the exact triggers for the onset and successful completion of the GABA-shift? The impact and participation of different cell types in the GABA-shift, and the ongoing maintenance of GABAergic signaling, and both intra- and extracellular chloride regulation is an extremely important topic, and the extent of the contribution of glial cells remains a topic of much interest.

## Author Contributions

KH and TC conceptualized and wrote the manuscript. YG and MT provided intellectual input and edited the manuscript. All authors contributed to the article and approved the submitted version.

## Conflict of Interest

The authors declare that the research was conducted in the absence of any commercial or financial relationships that could be construed as a potential conflict of interest.

## Publisher’s Note

All claims expressed in this article are solely those of the authors and do not necessarily represent those of their affiliated organizations, or those of the publisher, the editors and the reviewers. Any product that may be evaluated in this article, or claim that may be made by its manufacturer, is not guaranteed or endorsed by the publisher.
